# Novel III-V Nitride Polymorphs in the *P*4_2_/*mnm* and *Pbca* Phases

**DOI:** 10.3390/ma13173743

**Published:** 2020-08-24

**Authors:** Qingyang Fan, Xin Ai, Junni Zhou, Xinhai Yu, Wei Zhang, Sining Yun

**Affiliations:** 1 College of Information and Control Engineering, Xi’an University of Architecture and Technology, Xi’an 710055, China; ax_lee0219@163.com (X.A.); zjn@xauat.edu.cn (J.Z.); 2 Department of Mechanical and Electrical Engineering, Hetao College, Bayannur 015000, China; xhyu@stu.xidian.edu.cn; 3 School of Microelectronics, Xidian University, Xi’an 710071, China; wzhang-1993@stu.xidian.edu.cn; 4 Functional Materials Laboratory (FML), School of Materials Science and Engineering, Xi’an University of Architecture and Technology, Xi’an 710055, China

**Keywords:** III-V nitride polymorphs, direct band gap, stability, mechanical anisotropy

## Abstract

In this work, the elastic anisotropy, mechanical stability, and electronic properties for *P*4_2_/*mnm* XN (XN = BN, AlN, GaN, and InN) and *Pbca* XN are researched based on density functional theory. Here, the XN in the *P*4_2_/*mnm* and *Pbca* phases have a mechanic stability and dynamic stability. Compared with the *Pnma* phase and *Pm*-3*n* phase, the *P*4_2_/*mnm* and *Pbca* phases have greater values of bulk modulus and shear modulus. The ratio of the bulk modulus (*B*), shear modulus (*G*), and Poisson’s ratio (*v*) of XN in the *P*4_2_/*mnm* and *Pbca* phases are smaller than those for *Pnma* XN and *Pm*-3*n* XN, and larger than those for c-XN, indicating that *Pnma* XN and *Pm*-3*n* XN are more ductile than *P*4_2_/*mnm* XN and *Pbca* XN, and that c-XN is more brittle than *P*4_2_/*mnm* XN and *Pbca* XN. In addition, in the *Pbca* phases, XN can be considered a semiconductor material, while in the *P*4_2_/*mnm* phase, GaN and InN have direct band-gap, and BN and AlN are indirect wide band gap materials. The novel III-V nitride polymorphs in the *P*4_2_/*mnm* and *Pbca* phases may have great potential for application in visible light detectors, ultraviolet detectors, infrared detectors, and light-emitting diodes.

## 1. Introduction

Materials are the basis of human society and guide its development. The discovery and application of every major new material has brought changes to human life. Semiconductor materials are the key factor affecting the development of the semiconductor industry, and they have a vital strategic position. In the middle of the last century, the invention of single-crystal silicon and semiconductor transistors, and the successful development of their silicon-integrated circuits led to a revolution. Since the early 1970s, the successful development of quartz optical fiber materials and optical fibers—materials such as BN, BAs, and other III-V nitrides—and the invention of semiconductor lasers promoted the rapid development of optical fiber communication technology and gradually formed the high-tech industry.

In recent years, novel semiconductor materials have attracted an increasing number of researchers in materials research, such as carbon allotropes [1,2,3,4,5,6,7,8,9], light element compounds [10,11,12], Group IV semiconductor materials and theirs alloys [13,14,15,16,17,18,19,20], and III-V compounds [21,22,23,24,25,26,27,28,29,30,31,32,33,34,35]; Among them, III-V compounds have drawn much attention [21,22,23,24,25,26,27,28,29,30,31,32,33,34,35]. Dai et al. [21] studied type-II BN materials, which are mechanically and thermally stable at a temperature of 1000 K, and their large pore characteristics make them suitable for hydrogen storage. Miao et al. [22] investigated the structural, mechanical stability, and electronic properties of *P*6_4_22 AlP, GaP, and InP based on density functional theory (DFT) [36,37], as well as the thermal properties and elastic anisotropy. *P*6_4_22-AlP, *P*6_4_22-GaP, and *P*6_4_22-InP are dynamically and thermodynamically stable; *P*6_4_22-AlP and *P*6_4_22-GaP exhibit indirect band gaps, while *P*6_4_22-InP has a direct band gap of 0.42 eV and may be applied in infrared detectors. The thermodynamic and elastic properties of *β*-GaN semiconductors were studied, in detail, using DFT under an ultrasoft pseudopotential scheme by Fan et al. [25]. *β*-GaN has mechanical stability under a high pressure and temperature, and its *v*, *G*, *E*, and Zener anisotropy index show a greater anisotropy at a high temperature. Recently, Zhang et al. [26] also proposed III-nitride polymorphs *Pm*-3*n* XN (XN = BN, AlN, GaN, and InN) and studied their structure, mechanical properties, and electronic properties. Xiong et al. [27] investigated a *sp*^2^ + *sp*^3^ hybrid metal monoclinic 3D BN structure, and donated *M*-BN. In terms of energy, amid the metal BN structures predicted thus far, *M*-BN is the most favorable structure, and it is a potential new one-dimensional material, conductive along the *y*-direction. Additionally, the Vickers hardness of *M*-BN is 33.7 GPa, indicating that it has the potential to become a hard material.

The invention of the blue light diode won a Nobel Prize in physics. In order to make a blue light diode, another element needs to be doped in gallium nitride. If another phase GaN or another semiconductor material with a band gap can be achieved matching the blue light photon energy, steps can be saved in doping. In this work, based on DFT, the III-V nitride polymorphs XN (X = B, Al, Ga, and In) in *P*4_2_/*mnm* and *Pbca* phases are investigated. *P*4_2_/*mnm* phase GaN can absorb about 484 nm blue light, so it is a potential binary semiconductor material that can be directly made into a blue light diode. In addition, the stability and physical properties of the *P*4_2_/*mnm* XN and *Pbca* XN semiconductor materials are estimated and analyzed in this work.

## 2. Theoretical Methods 

DFT is utilized for the theoretical investigations, and all of the phonon spectra of *P*4_2_/*mnm* XN and *Pbca* XN are estimated according to the density functional perturbation theory (DFPT) method [38]. In the physical performance prediction and structural geometry optimization calculations, the Perdew–Burke–Ernzerhof (PBE) functional of the exchange-correlation potential and the generalized gradient approximation (GGA) [39] were used, and the DFT calculations were carried out using the ultrasoft pseudopotentials [40] from the Cambridge Sequential Total Energy Package (CASTEP) code [41]. The valence electron structures of the N, B, Al, Ga, and In atoms were 2*s*^2^2*p*^3^, 2*s*^2^2*p*^1^, 3*s*^2^3*p*^1^, 4*s*^2^4*p*^1^, and 5*s*^2^5*p*^1^, respectively. A high *k*-point separation, less than or approximately 0.025 Å^−1^ × 2π, was used in the *P*4_2_/*mnm* XN and *Pbca* XN, and 9 × 9 × 16, 8 × 8 × 13, 7 × 7 × 12, and 6 × 6 × 11 Monkhorst–Pack meshes [42] were adopted for the conventional cells of XN (X = B, Al, Ga, and In) in the *P*4_2_/*mnm* phase, and 8 × 8 × 9, 6 × 7 × 8, 6 × 7 × 7, and 6 × 6 × 7 Monkhorst–Pack meshes were adopted for the conventional cells of XN in the *Pbca* phase, respectively. The geometric optimizations were performed using the Broyden–Fletcher–Goldfarb–Shanno (BFGS) minimization scheme [43]. Additionally, in order to make the energy of the new prediction structure converge and be in the lowest energy state (with a total energy difference between the two times being less than 0.001 eV), we used 500 eV of plane wave cut-off energy for the structural optimization and performance investigation of XN in the *P*4_2_/*mnm* and *Pbca* phases, respectively. The electronic energy band structures were estimated using the Heyd–Scuseria–Ernzerhof (HSE06) hybrid functional [44]. 

## 3. Results and Discussion

### 3.1. Structural Properties

**Figure 1:**
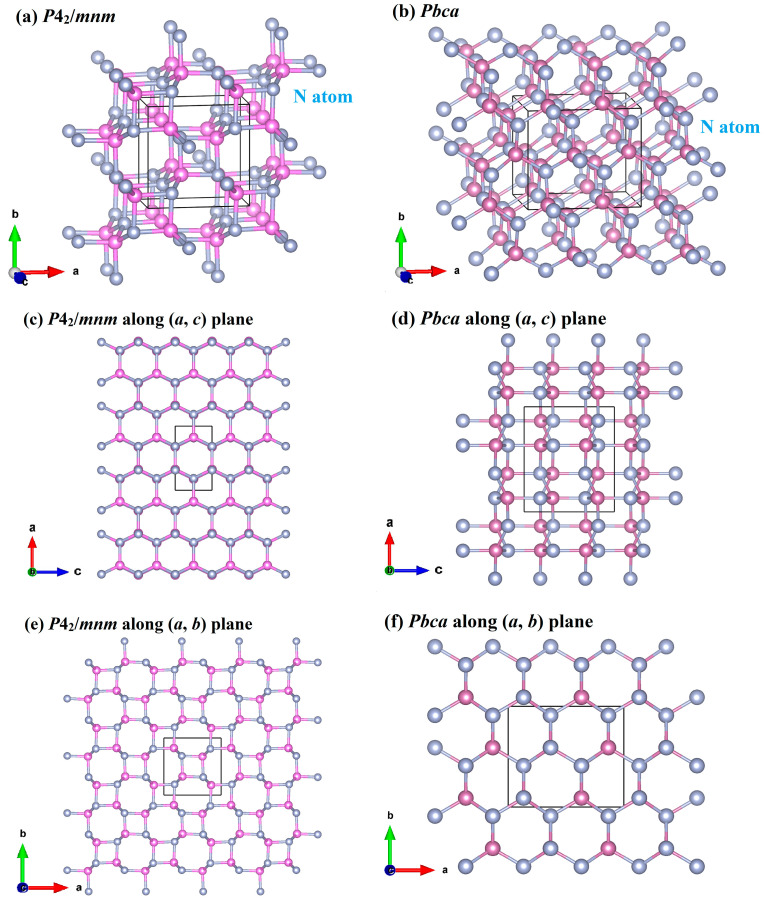
Crystal structure of XN in the *P*4_2_/*mnm* phase (**a**) and the *P*4_2_/*mnm* phase along the *ac* plane (**c**) and the *ab* plane (**e**); crystal structures of XN in the *Pbca* phase (**b**) and the *Pbca* phase along the *ac* plane (**d**) and the *ab* plane (**f**).

The crystal structures of XN in the *P*4_2_/*mnm* and *Pbca* phases are illustrated in Figure 1. Blue spheres indicate nitrogen atoms; violet spheres indicate B, Al, Ga, and In atoms, because the four varieties of atoms are in the same positions; and black regions indicate the conventional cell. Both of the crystal structures of *P*4_2_/*mnm* XN and *Pbca* XN are composed of *sp*^3^-bonded rings, in which there are four-, six-, and eight-membered X-N rings in the *P*4_2_/*mnm* phase (Figure 1a,e), and six-membered rings in the *Pbca* phase (Figure 1b). Figure 1e shows that in the *P*4_2_/*mnm* phase, the X atoms and nitrogen atoms are alternately connected along the *ab* plane to form four-membered rings and eight-membered rings. It can be found from Figure 1c, that the structure of the *P*4_2_/*mnm* XN along the *ac* plane and the structure of *Pbca* XN along the *ab* plane are six-membered rings composed of alternately appearing X atoms and nitrogen atoms.

The calculated lattice parameters of the *P*4_2_/*mnm* XN, *Pbca* XN, *Pm-*3*n* XN, and *Pnma* XN are presented in Table 1, where *a*, *b*, and *c* express the lattice parameters, *ρ* represents the density, and *V* represents the conventional volume. The obtained lattice parameters in the *P*4_2_/*mnm* phase and *Pbca* phase gradually increased from BN to InN. In the *P*4_2_/*mnm* and *Pbca* phase, *ρ* and *V* gradually increased from BN to AlN along the III-V nitrides because of the atomic radius and relative atomic mass of the X atom increasing gradually. Accordingly, the volume of the XN f.u. (formular unit) in the *Pbca* phase was greater than that in *P*4_2_/*mnm* phase, so the *ρ* of *Pbca* XN was slightly smaller than that of the *P*4_2_/*mnm* XN. What is more, the densities of *P*4_2_/*mnm* XN and *Pbca* XN were higher than that of *Pm*-3*n* XN and *Pnma* XN, this is also due to the increasing atomic radius and the relative atomic mass of the X atom. It can be found from Table 1 that the crystal volumes of *P*4_2_/*mnm* XN, *Pbca* XN, *Pm*-3*n* XN, and *Pnma* XN from large to small followed the order of *Pm*-3*n* XN, *Pnma* XN, *Pbca* XN, *P*4_2_/*mnm* XN, and c-XN.

### 3.2. Stability

**Figure 2:**
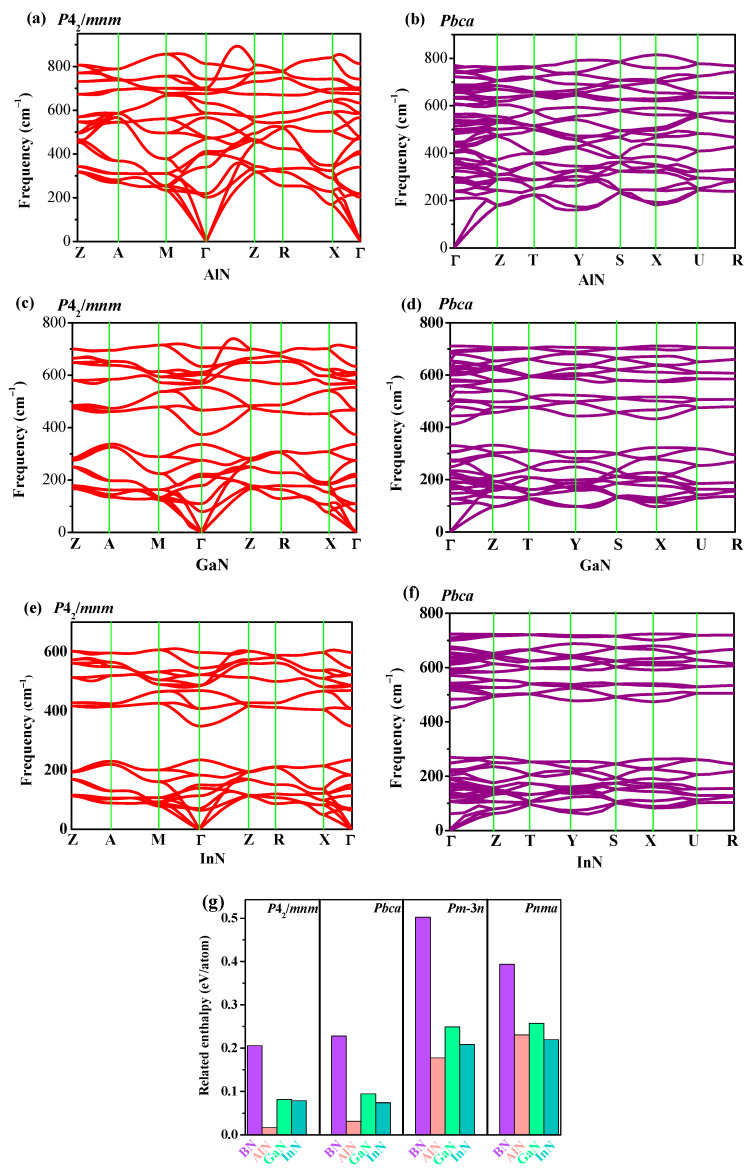
Phonon dispersion branches of AlN (**a**), GaN (**c**), and InN (**e**) in the *P*4_2_/*mnm* phase, phonon dispersion branches of AlN (**b**), GaN (**d**), and InN (**f**) in the *Pbca* phase, and related enthalpies (**g**) of XN in the *P*4_2_/*mnm*, *Pbca*, *Pm*-3*n* and *Pnma* phases.

Stability is an important physical property of materials. To study the dynamic stability of *P*4_2_/*mnm* XN and *Pbca* XN, the phonon spectra of AlN, GaN, and InN in the *P*4_2_/*mnm* and *Pbca* phases are presented in Figure 2a–f, respectively. There were no phonons with imaginary frequencies observed in whole Brillouin zone, which proves that aluminum nitride, gallium nitride, and indium nitride in the *P*4_2_/*mnm* and *Pbca* phases are all dynamically stable. The frequencies of all of the optical phonons at the Brillouin zone center (Gamma-point) of the *P*4_2_/*mnm* phase and *Pbca* phase are given in Tables S1 and S2 in Supporting Information, respectively. Whether the material can be synthesized was predicted by analyzing the enthalpy. The related enthalpies of XN in the *P*4_2_/*mnm*, *Pbca*, *Pm-*3*n,* and *Pnma* phases are presented in Figure 2g.

The relative enthalpies of XN in the *P*4_2_/*mnm*, *Pbca*, *Pm-*3*n,* and *Pnma* phases were be obtained using the following: *ΔH* = *H_novel phase_/m-H_c-XN phase_/n;* here, *m* and *n* are the amount of BN in the conventional cell. From Figure 2g, all of the enthalpies of XN in the *P*4_2_/*mnm* phase and *Pbca* phase XN were less than those in the *Pm-*3*n* and *Pnma* phases, which indicates that they were more favorable than the *Pm-*3*n* XN and *Pnma* XN theoretically, which could be synthesized in experiments. The two new XN phases proposed in this work compared the relative enthalpy of *Pbca* XN (XN = BN, AlN, and GaN) with that of *P*4_2_/*mnm* XN (XN = BN, AlN, GaN), and it could be found that the relative enthalpy of *Pbca* XN was slightly larger, while the *Pbca* InN was more favorable than *P*4_2_/*mnm* InN. The *P*4_2_/*mnm* phase belongs to the tetragonal system, and the Born’s mechanical stability criterion for the tetragonal system can be given as the following equations [49]:

(1) $$C_{11}>0,C_{33}>0,C_{44}>0,C_{66}>0,$$

(2) $$(C_{11}-C_{12})>0,(C_{11}+C_{33}-2C_{13})>0,$$

(3) $$[2(C_{11}+C_{12})+C_{33}+4C_{13}]>0.$$

The *Pbca* phase belongs to the orthorhombic system, and the Born’s criterion for the orthorhombic system can be indicated by the following equations [49]:

(4) $$C_{11}>0,C_{11}C_{22}>C_{12}^{2},$$

(5) $$C_{11}C_{22}C_{33}+2C_{12}C_{13}C_{23}-C_{11}C_{23}^{2}-C_{22}C_{13}^{2}-C_{33}C_{12}^{2}>0,$$

(6) $$C_{44}>0,C_{55}>0,C_{66}>0.$$

The calculated elastic constants of XN in the *P*4_2_/*mnm*, *Pbca*, *Pm*-3*n*, *Pnma* phases and c-XN are listed in Table 2. The theoretical data of c-XN calculated in this work are excellent agreement with the experimental results and theoretical results reported before [50,51,52,53]. From Table 2, one can see that the elastic constants of XN in the *P*4_2_/*mnm* phase and *Pbca* phase satisfy the above Born criterion, thus proving that *P*4_2_/*mnm* XN and *Pbca* XN are both mechanically stable. The synthesis methods of these materials can refer to the calculations from References [54,55,56]. These are the structures grown on sapphire substrates. By selecting suitable substrates and matching lattice parameters, new predicted phases may be expected to be grown by MOVPE or MOCVD (Metal-organic Vapor Phase Epitaxy or Metal-organic Chemical Vapor Deposition).

### 3.3. Mechanical and Anisotropy Properties

The Young’s modulus (*E*), bulk modulus (*B*), shear modulus (*G*), the ratio of *B* to *G*, and Poisson’s ratio (*v*) of III-V nitrides from boron to indium in the *P*4_2_/*mnm*, *Pbca*, *Pnma*, *Pm*-3*n* and cubic phases are listed in Table 2. The vales of *B* and *G* are estimated using the Voigt–Reuss–Hill approximations [57,58,59]. For a tetragonal system, the following equations are used [53]:

(7) $$B_{v}=(1/9)[2(C_{11}+C_{12})+C_{33}+4C_{13}],$$

(8) $$G_{V}=(1/30)(M+3C_{11}-3C_{12}+12C_{44}+6C_{66}),$$

(9) $$B_{R}=C^{2}/M,$$

(10) $$G_{R}=15{\left\{(18B_{V}/C^{2})+[6/(C_{11}-C_{12})]+(6/C_{44})+(3/C_{66})\right\}}^{-1},$$

(11) $$M=C_{11}+C_{12}+2C_{33}-4C_{13},$$

(12) $$C^{2}=(C_{11}+C_{12})C_{33}-2C_{13}^{2}.$$

For an orthorhombic system, the following equations are used [53]:

(13) $$B_{V}=(1/9)[C_{11}+C_{22}+C_{33}+2(C_{12}+C_{13}+C_{23})],$$

(14) $$G_{V}=(1/15)[C_{11}+C_{22}+C_{33}+3(C_{44}+C_{55}+C_{66})-(C_{12}+C_{13}+C_{23})],$$

(15) $$\begin{array}{ll} B_{R}= & \Delta [C_{11}(C_{22}+C_{33}-2C_{23})+C_{22}(C_{33}-2C_{13})-2C_{33}C_{12}\\ & +C_{12}(2C_{23}-C_{12})+C_{13}(2C_{12}-C_{13})+C_{23}(2C_{13}-C_{23}){]}^{-1}, \end{array}$$

(16) $$\begin{array}{ll} G_{R} & =15\{4[C_{11}(C_{22}+C_{33}+C_{23})+C_{22}(C_{33}+C_{13})+C_{33}C_{12}\\ & -C_{12}(C_{23}+C_{12})-C_{13}(C_{12}+C_{13})-C_{23}(C_{13}+C_{23})]/\Delta \\ & +3[(1/C_{44})+(1/C_{55})+(1/C_{66})]{\}}^{-1}, \end{array}$$

(17) $$\Delta =C_{13}(C_{12}C_{23}-C_{13}C_{22})+C_{23}(C_{12}C_{13}-C_{23}C_{11})+C_{33}(C_{11}C_{22}-C_{12}^{2}).$$

The values of *B*, *G*, *E,* and *v* of the *P*4_2_/*mnm* XN and *Pbca* XN are calculated by the following:

(18) $$B=\frac{1}{2}(B_{V}+B_{R}),G=\frac{1}{2}(G_{V}+G_{R}),$$

(19) E=9BG/(3B+G),v=(3B−2G)/[2(3B+G)].

**Figure 3:**
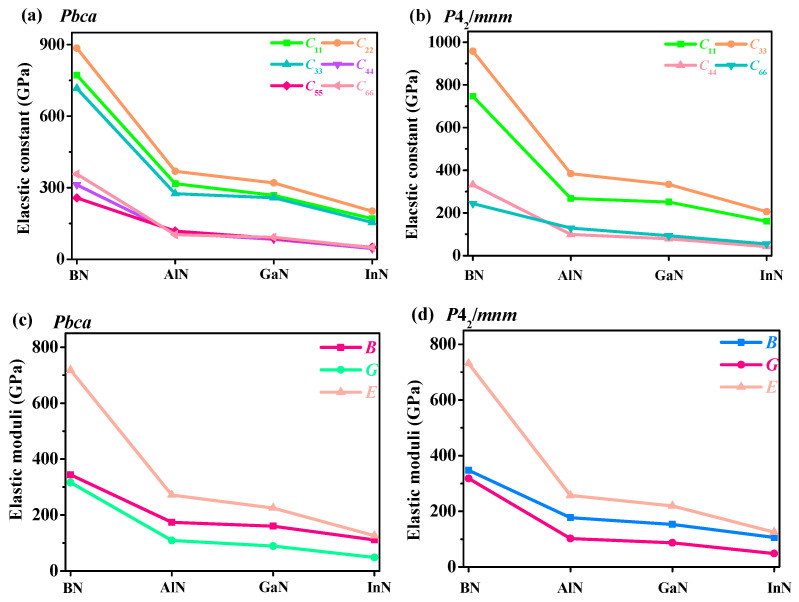
Elastic constants and elastic moduli of the *Pbca* XN (**a**,**c**) and *P*4_2_/*mnm* XN (**b**,**d**).

From Table 2, all of the elastic moduli of *P*4_2_/*mnm* XN and *Pbca* XN were greater than those of *Pm*-3*n* XN and *Pnma* XN, but less than those of c-XN. In contrast, the *B/G* and *v* of *P*4_2_/*mnm* XN and *Pbca* XN were smaller than those of *Pm*-3*n* XN and *Pnma* XN, but greater than those of c-XN. It is obvious that the elastic constants, *C*_11_, *C*_22_, *C*_33_, *C*_44_, *C*_55_, and *C*_66_, and elastic moduli, *B*, *G*, and *E,* of the III-V compounds reduced from the boron atom to indium atom, while the value of *v* gradually increased in the *Pbca* and *P*4_2_/*mnm* phases in Figure 3. Young’s modulus can be regarded as an index to measure the difficulty of elastic deformation. The larger the Young’s modulus, the less likely it is to deform. In the *Pbca* phase and *P*4_2_/*mnm* phase, the *B*, *G*, and *E* of BN were the largest, and those of InN were the smallest, which indicates that BN is more difficult to deform than other III-V nitrides. In addition, in the *Pbca* phase, the elastic moduli of BN were slightly smaller than those in the *P*4_2_/*mnm* phase, whereas the *B* and *E* of *Pbca* InN were slightly higher than those of *P*4_2_/*mnm* InN.

**Figure 4:**
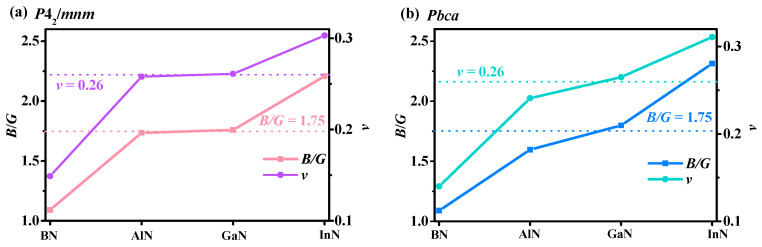
Bulk modulus (*B*) and shear modulus (*G*) ratio (*B*/*G*) and Poisson’s ratio (*v*) of *P*4_2_/*mnm* XN (**a**), and *B*/*G* and *v* of *Pbca* XN (**b**).

Shear modulus is a mechanical property of materials, which characterizes the resistance to plastic deformation of materials. The bulk modulus of the crystal is the macroscopic property of the material, which reflects the resistance of the material to the uniform compression of the outside under the elastic system. Therefore, in order to investigate the brittleness and ductility of materials, the *B*/*G* of *Pbca* XN and *P*4_2_/*mnm* XN were analyzed. The values of the ratio of *B* to *G* and *v* of XN in the *Pbca* phase and *P*4_2_/*mnm* phase are plotted in Figure 4. Taking 1.75 as the dividing line, the *B*/*G* value exceeding 1.75 indicates ductility; otherwise, it indicates brittleness. In addition, by comparing the Poisson’s ratio (*v*) with other elastic constants, a great deal of information about bond strength characteristics can be obtained [60]. The difference between *v* and *B*/*G* is that 0.26 is used as the dividing line. From Figure 4, GaN and AlN in *P*4_2_/*mnm* phase and *Pbca* phase were very close to the boundary of *B*/*G* and *v*. The *B*/*G* and *v* for *P*4_2_/*mnm* AlN and *Pbca* AlN were both smaller than the dividing line, so they tended to be brittle, the *B*/*G* and *v* for *P*4_2_/*mnm* GaN and *Pbca* GaN were both larger than the dividing line, so they tended to be ductile. In addition, the ratio of the bulk modulus to shear modulus and the *v* increased from BN to InN in the *P*4_2_/*mnm* phase and *Pbca* phase; that is to say, BN and AlN are brittle materials, and GaN and InN are ductile materials. From Table 2, the *B*/*G* ratio and *v* of *P*4_2_/*mnm* XN and *Pbca* XN were smaller than those of *Pnma* XN, but higher than those of c-XN, indicating that *P*4_2_/*mnm* XN and *Pbca* XN are more brittle than *Pnma* XN, but weaker than c-XN.

**Figure 5:**
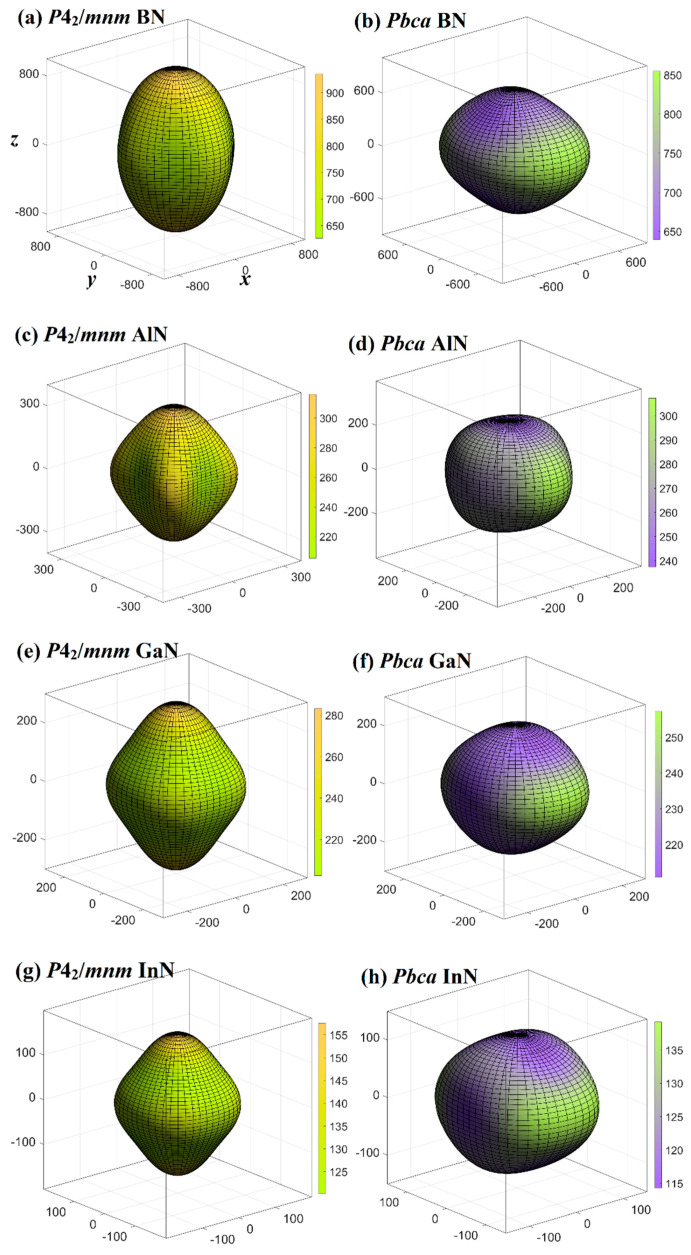
Three-dimensional contour plots of the Young’s modulus for BN (**a**), AlN (**c**), GaN (**e**), and InN (**g**) in the *P*4_2_/*mnm* phase; and three-dimensional contour plots of the Young’s modulus for BN (**b**), AlN (**d**), GaN (**f**), and InN (**h**) in the *Pbca* phase.

It is known that elastic anisotropy plays a significant role in researching the physical properties of crystals [31]. The three-dimensional (3D) contour can visually display the mechanical anisotropy, because for anisotropic materials, the values of *E* will change in distinct directions. If the three-dimensional contour is a regular sphere, then the mechanical properties of the material show isotropy; if it is not a regular sphere, then the material exhibits anisotropy of mechanical properties [61,62,63,64,65], and the lower the similarity to a sphere is, the greater the anisotropy. In order to figure out the elasticity anisotropy of Young’s modulus for XN in the *P*4_2_/*mnm* and *Pbca* phases in distinct directions, the variations of *E* for *P*4_2_/*mnm* XN and *Pbca* XN in different directions are illustrated in Figure 5. As shown in Figure 5, the values of *E*_max_ and *E*_min_ in the *P*4_2_/*mnm* phase are represented by the curved surface of the yellow and the green line, respectively, and in the *Pbca* phase they are represented by the curved surface of the green and blue line, respectively. The maximum, minimum, and maximum/minimum ratios of *P*4_2_/*mnm* XN and *Pbca* XN for Young’s modulus, which are marked as *E*_max_, *E*_min_, and *E*_max_/*E*_min_, respectively. For XN in the *Pbca* phase, the maximum/minimum ratios of Young’s modulus were 1.34, 1.29, 1.22, and 1.22, respectively, which were smaller than those in the *P*4_2_/*mnm* phase (the *E*_max_/*E*_min_ of *P*4_2_/*mnm* XN were 1.49, 1.54, 1.40 and 1.31, respectively.). That is, the elastic anisotropy of *E* decreased in the *Pbca* phase, in which the Young’s modulus of *Pbca* BN had the largest elastic anisotropy and *Pbca* InN had the smallest elastic anisotropy, and the elastic anisotropy of *E* for *Pbca* XN was smaller than that of *P*4_2_/*mnm* XN. The anisotropy in the *Pbca* phase was less anisotropic than in the other phases, which can be considered to be from the following aspects. The first factor to be considered is that the constituent elements of each phase are all XN—they all have only X and nitrogen elements. The second factor to be considered is the length of the chemical bond, the bond lengths in different phases are all X-N bond lengths; the bond lengths of all X-N are listed in Cif files in Supporting information. The third aspect to consider is the type of chemical bond, they are all *sp^3^* hybrids; both X and nitrogen atoms are coordinated in the *Pbca* phase, *P*4_2_/*mnm* phase, *Pm*-3*n* phase, and *Pnma* phase. It can be concluded that in the *P*4_2_/*mnm* phase, the anisotropy of AlN was the largest, and that of InN was the smallest. Compared with XN in *P*4_2_/*mnm* phase, the XN in the *Pbca* phase had less anisotropy from BN to InN; their 3D contours were obviously closer to a sphere in Figure 5. It is worth noting that in the *P*4_2_/*mnm* phase and *Pbca* phase, the three-dimensional contour from BN to AlN, GaN, and finally InN became closer to a sphere. In other words, the elastic anisotropy of the Young’s modulus of the XN *P*4_2_/*mnm* phase and *Pbca* phase gradually decreased.

### 3.4. Electronic Properties

**Figure 6:**
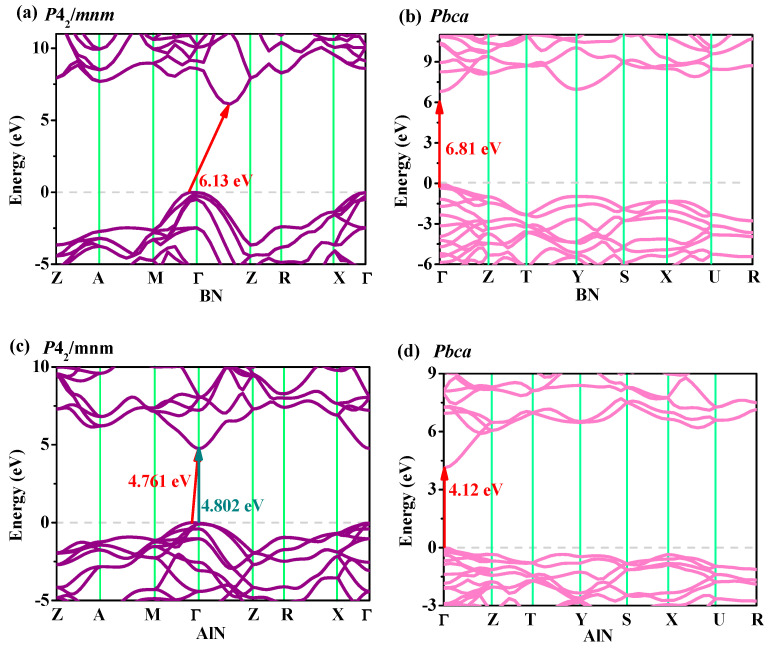

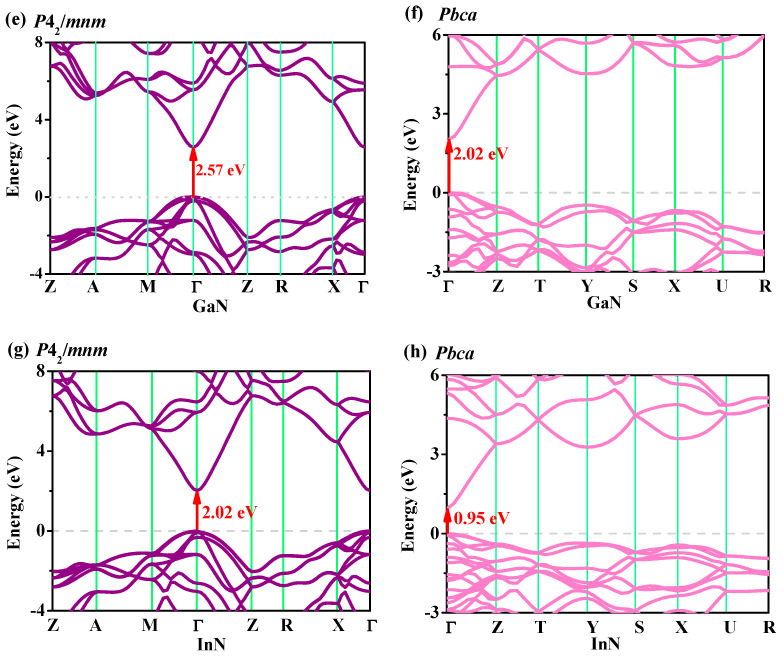
Electronic band structures for BN (**a**), AlN (**c**), GaN (**e**), and InN (**g**) in the *P*4_2_/*mnm* phase; and electronic band structures for BN (**b**), AlN (**d**), GaN (**f**), and InN (**h**) in the *Pbca* phase.

The electronic band structure of materials is often used to describe the prohibited or permitted energy of electrons. An analysis of the band structure can reveal more properties in the field of electronics and optics. The energy band structures of XN in the *P*4_2_/*mnm* phase and *Pbca* phase are illustrated in Figure 6. From Figure 6a–f, *P*4_2_/*mnm* XN and *Pbca* XN can be used as semiconductor materials. *Pbca* XN belongs to the direct band gap materials, and the band gaps of *P*4_2_/*mnm* GaN and *P*4_2_/*mnm* InN had direct band gap materials of 2.57 eV and 2.02 eV, respectively. The BN and AlN in the *P*4_2_/*mnm* phase had an indirect and wide band gap of 6.13 eV and 4.76 eV, respectively, but the indirect band gap in *P*4_2_/*mnm* AlN was very close to that of the direct band gap, so the *P*4_2_/*mnm* AlN belonged to quasi-direct band gap semiconductor materials. For *P*4_2_/*mnm* AlN, the conduction band minimum (CBM) was located at Γ (0.0000, 0.0000, 0.0000), while the valence band maximum (VBM) was located at (0.0769, 0.0769, 0.0000) along the M–Γ direction (see Figure 6c); it had a direct band gap of 4.802 eV, it was only 0.041 eV different from the indirect band gap. The band gaps of GaN, AlN, and InN in the *P*4_2_/*mnm* phase were marginally larger than in the *Pbca* phase. The same compounds had different band gaps in the different phases. For the III-V nitrides, the band gap range in the *P*4_2_/*mnm* phase (2.02–6.13 eV) was slightly smaller than those in the *Pbca* (0.95–6.81 eV), *Pm*-3*n* (1.04–5.87 eV) [26], and *Pnma* (0.66–7.18 eV) phases [28,29]. In the *P*4_2_/*mnm*, *Pbca*, *Pm*-3*n,* and *Pnma* phases, *Pnma* BN had the largest band gap, while *Pnma* InN had the smallest band gap, indicating that the *Pnma* phase had the largest band gap range. By doping impurities between X atoms or P atoms, the energy band gap could be adjusted, and ternary, quaternary, or higher compounds could be formed. For example, doping impurity Ga atoms into InN in the *P*4_2_/*mnm* phase could form a ternary compound with a band gap of 2.02 to 2.57 eV. In general, both *P*4_2_/*mnm* XN and *Pbca* XN had wide band gaps in the range of 2.02–6.13 eV and 0.95–6.81 eV, respectively. Theoretically, these compounds may have good application prospects in the production of photoelectric semiconductor devices, for example, as blue light emitting diodes (GaN in *P*4_2_/*mnm* phase) and orange light emitting diodes (GaN in *Pbca* phase and InN in *P*4_2_/*mnm* phase).

## 4. Conclusions

In this manuscript, the structural, stability, anisotropy, and mechanical properties of XN in the *P*4_2_/*mnm* and *Pbca* phases are presented. According to the phonon dispersion branches and elastic constants, *P*4_2_/*mnm* XN (XN = BN, AlN, GaN, and InN) and *Pbca* XN (XN = BN, AlN, GaN, and InN) are stable. Among the *P*4_2_/*mnm* phase and *Pbca* phase, BN has the strongest brittleness, while InN has the strongest ductility. In addition, the *E* and *B* of *P*4_2_/*mnm* XN and *Pbca* XN are greater than those of *Pm*-3*n* XN and *Pnma* XN. The elastic anisotropy of *E* decreases gradually as the group III-V nitrides change from BN to InN in the *P*4_2_/*mnm* phase and *Pbca* phase, and it is smaller than that of c-XN. The structures of the electronic band of *Pbca* XN (XN = BN, AlN, GaN, and InN), *P*4_2_/*mnm* GaN, and *P*4_2_/*mnm* InN indicate that they can be candidates for semiconductor materials, and they have direct band gaps within HSE06, while BN and Al in the *P*4_2_/*mnm* phase are semiconductor materials with an indirect and wide band gap. Moreover, for the XN (XN = BN, AlN, GaN, and InN) compounds, the band gap range of the *P*4_2_/*mnm* phase (2.02–6.13 eV) is slightly smaller than those of the *Pbca* (0.95–6.81 eV), *Pnma* (0.66–7.18 eV), and *Pm*-3*n* (1.04–5.87 eV) phases. These compounds may have good application prospects in the electronics manufacturing industry.

## Figures and Tables

**Table 1:** Lattice parameters and volumes of *Pbca* XN, *P*4_2_/*mnm* XN, c-XN, *Pm*-3*n* XN, wurtzite XN, and *Pnma* XN.

Materials	Methods	*a*	*b*	*c*	*c/a*	*ρ*	*V*
*Pbca* BN	GGA	5.110	4.434	4.399		3.308	12.459
*Pbca* AlN	GGA	6.183	5.428	5.291		3.067	22.192
*Pbca* GaN	GGA	6.428	5.602	5.518		5.599	24.837
*Pbca* InN	GGA	7.172	6.262	6.150		6.196	34.525
*P*4_2_/*mnm* BN	GGA	4.421		2.548		3.309	12.203
*P*4_2_/*mnm* AlN	GGA	5.320		3.116		3.087	22.049
*P*4_2_/*mnm* GaN	GGA	5.547		3.221		5.612	24.779
*P*4_2_/*mnm* InN	GGA	6.184		3.608		6.202	34.493
c-BN	GGA	3.623					11.884
	LDA	3.576					11.436
	Exp. ^a^	3.620					
c-AlN	GGA	4.396				3.206	21.229
c-GaN	GGA	4.557				5.878	23.658
	LDA	4.523					23.132
	Exp. ^b^	4.520					23.332
c-InN	GGA	5.088				6.496	32.932
*Pm*-3*n* BN	GGA ^c^	4.438				2.829	14.569
*Pm*-3*n* AlN	GGA ^c^	5.366				2.643	25.751
*Pm*-3*n* GaN	GGA ^c^	5.584				4.793	29.015
*Pm*-3*n* InN	GGA ^c^	6.237				5.291	40.428
*Pnma* BN	GGA ^d^	4.890	2.589	4.284		3.040	13.557
*Pnma* AlN	GGA ^e^	5.775	3.198	5.123		2.828	24.068
*Pnma* GaN	GGA ^e^	6.076	3.302	5.421		5.114	27.190
*Pnma* InN	GGA ^e^	6.777	3.699	6.051		5.642	37.918
wurtzite BN	GGA	2.539		4.200	1.654		
	Exp. ^f^	2.550		4.199	1.647		
wurtzite AlN	GGA	3.103		5.010	1.615		
	Exp. ^g^	3.110		4.980	1.601		
wurtzite GaN	GGA	3.186		5.223	1.639		
	Exp. ^g^	3.190		5.200	1.630		
wurtzite InN	GGA	3.547		5.726	1.614		
	Exp. ^g^	3.540		5.710	1.613		

^a^ Ref. [45], ^b^ Ref. [46], ^c^ Ref. [26], ^d^ Ref. [28], ^e^ Ref. [29]. ^f^ Ref. [47], ^g^ Ref. [48].

**Table 2:** Elastic constants (GPa) and elastic moduli (GPa) of *Pbca* XN, *P*4_2_/*mnm*, c-XN, *Pm*-3*n* XN and *Pnma* XN.

	C_11_	C_12_	C_13_	C_22_	C_23_	C_33_	C_44_	C_55_	C_66_	B	G	B/G	E	v
*Pbca* BN	772	135	139	885	92	716	312	257	357	344	316	1.09	718	0.140
*Pbca* AlN	317	122	92	369	94	275	109	118	103	174	109	1.60	271	0.241
*Pbca* GaN	268	117	92	320	91	258	84	88	92	160	89	1.80	225	0.265
*Pbca* InN	170	95	72	202	75	154	45	50	49	111	48	2.31	126	0.311
P4_2_/*mnm* BN	747	144	99	747		958	332		244	347	318	1.09	731	0.149
P4_2_/*mnm* AlN	268	116	114	268		384	99		129	177	102	1.74	257	0.258
P4_2_/*mnm* GaN	251	90	93	251		334	79		93	153	87	1.76	219	0.261
P4_2_/*mnm* InN	161	59	80	161		206	42		55	106	48	2.21	125	0.303
c-BN	805	187					468			393	396	0.99	889	0.123
	820^a^	190					480			400				
c-AlN	280	146					184			190	123	1.54	304	0.234
	294^b^	160					189							
c-GaN	247	127					150			167	104	1.61	258	0.242
	263^c^	145					156			184	106	1.74	267	0.258
	285^d^	161					149			202^e^	105^e^			
c-InN	152	95					86			114	55	2.07	142	0.292
*Pm*-3*n* BN	700 ^f^	85					209			290	244	1.19	572	0.171
*Pm*-3*n* AlN	335 ^f^	59					58			151	83	1.82	210	0.268
*Pm*-3*n* GaN	238 ^f^	61					58			120	69	1.74	174	0.259
*Pm*-3*n* InN	173 ^f^	55					36			95	44	2.16	114	0.299
*Pnma* BN	392 ^g^	99	256	770	116	675	299	272	187	298	227	1.31	543	0.196
*Pnma* AlN	160 ^h^	68	133	332	137	282	92	118	82	149	80	1.86	204	0.272
*Pnma* GaN	152 ^h^	59	116	278	119	267	68	93	67	133	69	1.93	176	0.279
*Pnma* InN	109 ^h^	48	81	173	92	165	34	52	38	94	38	2.47	100	0.322

^a^ Ref. [50]-Experimental, ^b^ Ref. [51]-Experimental, ^c^ Ref. [25], ^d^ Ref. [52], ^e^ calculated by *B*= (*B*_V_ + *B*_R_)/2, *B*_V_ = *B*_R_ = (*C*_11_ + 2*C*_12_)/3, *G* = (*G*_V_ + *G*_R_)/2, *G*_V_ = (*C*_11_ − *C*_12_ + 3*C*_44_)/5, and *G*_R_ = 5(*C*_11_ − *C*_12_)*C*_44_/[4*C*_44_ + 3(*C*_11_ − *C*_12_)], for Cubic system in Ref. [53]; ^f^ Ref. [26], ^g^ Ref. [28], ^h^ Ref. [29].

## Supplementary Materials

The following are available online at https://www.mdpi.com/1996-1944/13/17/3743/s1, Table S1: The frequencies of all optical phonons (cm^−1^) at the Brillouin Zone center (Gamma-point) of *P*4_2_/*mnm* phase; Table S2: The frequencies of all optical phonons (cm^−1^) at the Brillouin Zone center (Gamma-point) of *Pbca* phase.
