# Supplementary material for: Novel III-V Nitride Polymorphs in the P42/mnm and Pbca Phases
Source: Materials (Basel). 2020 Aug 24;13(17):3743. doi: 10.3390/ma13173743 (PMC7504143; doi:10.3390/ma13173743)
Supplement: Supplementary file 1 [file materials-13-03743-s001.pdf]

materials

Supplementary

Novel III-V Nitride Polymorphs in the $P4_2/mnm$ and *Pbca* Phases

Qingyang Fan [1,*], Xin Ai [1], Junni Zhou [1], Xinhai Yu [2], Wei Zhang [3] and Sining Yun [4,*]

[1] College of Information and Control Engineering, Xi'an University of Architecture and Technology, Xi'an 710055, China; ax_lee0219@163.com (X.A.); zjn@xauat.edu.cn (J.Z.)
[2] Department of Mechanical and Electrical Engineering, Hetao College, Bayannur 15000, China; xhyu@stu.xidian.edu.cn
[3] School of Microelectronics, Xidian University, Xi'an 710071, China; wzhang-1993@stu.xidian.edu.cn
[4] Functional Materials Laboratory (FML), School of Materials Science and Engineering, Xi'an University of Architecture and Technology, Xi'an 710055, China;
* Correspondence: fanqy@xauat.edu.cn; (Q.F.); yunsining@xauat.edu.cn (S.Y.)

Received: 8 July 2020; Accepted: 17 August 2020; Published: date

Abstract: In this work, the elastic anisotropy, mechanical stability, and electronic properties for $P4_2/mnm$ XN (XN = BN, AlN, GaN, and InN) and *Pbca* XN are researched based on density functional theory. Here, the XN in the $P4_2/mnm$ and *Pbca* phases have a mechanic stability and dynamic stability. Compared with the *Pnma* phase and *Pm*-3*n* phase, the $P4_2/mnm$ and *Pbca* phases have greater values of bulk modulus and shear modulus. The ratio of the bulk modulus (B), shear modulus (G), and Poisson's ratio (v) of XN in the $P4_2/mnm$ and *Pbca* phases are smaller than those for *Pnma* XN and *Pm*-3*n* XN, and larger than those for c-XN, indicating that *Pnma* XN and *Pm*-3*n* XN are more ductile than $P4_2/mnm$ XN and *Pbca* XN, and that c-XN is more brittle than $P4_2/mnm$ XN and *Pbca* XN. In addition, in the *Pbca* phases, XN can be considered a semiconductor material, while in the $P4_2/mnm$ phase, GaN and InN have direct band-gap, and BN and AlN are indirect wide band gap materials. The novel III-V nitride polymorphs in the $P4_2/mnm$ and *Pbca* phases may have great potential for application in visible light detectors, ultraviolet detectors, infrared detectors, and light-emitting diodes.

Keywords: III-V nitride polymorphs; direct band gap; stability; mechanical anisotropy

Table 1. The frequencies of all optical phonons (cm^{-1}) at the Brillouin Zone center (Gamma-point) of $P4_2/mnm$ phase.

	AlN	GaN	InN
1	201.5857	79.4292	63.6486
2	218.5225	110.0640	70.1519
3	339.9270	178.6611	112.9672
4	400.8122	213.6351	138.6136
5	412.1600	223.1330	150.1105
6	471.8027	275.2356	182.9804
7	475.8792	336.3222	234.4892
8	565.8705	373.5736	348.4896
9	586.8565	467.1092	407.4732
10	632.3637	554.4032	468.8439
11	675.7899	569.4227	484.2429
12	692.8340	577.7109	486.6782
13	694.4315	602.6127	522.3160
14	702.4293	611.5555	522.4219
15	742.0521	633.3643	544.4685
16	812.4631	704.9506	597.5544

Table 2. The frequencies of all optical phonons (cm^{-1}) at the Brillouin Zone center (Gamma-point) of *Pbca* phase.

	AlN	GaN	InN
1	5.4228	107.8685	3.4404
2	207.7010	126.6695	61.70629
3	239.7562	127.0121	106.9416
4	240.3222	148.3830	108.0202
5	277.8047	164.0547	121.5723
6	291.4199	174.1371	128.0684
7	294.9902	178.3036	140.2349
8	297.8589	186.6872	140.4762
9	324.8907	192.5885	148.9418
10	336.8508	206.9010	160.3257
11	354.5625	207.9852	170.7902
12	366.2182	221.0460	173.0551
13	372.3738	225.4155	189.4223
14	377.9194	234.1429	190.3757
15	402.3623	248.7290	192.9396
16	408.0920	270.5174	204.1058
17	429.6848	271.4617	216.6756
18	433.7901	276.4911	217.1729
19	437.8848	304.9743	223.8659
20	446.4661	306.8264	247.4741
21	481.8310	330.1116	248.6704
22	487.4944	412.0965	269.7590
23	499.9197	443.9439	449.8122
24	514.2899	461.0132	483.5194
25	529.3710	480.2074	516.7468
26	538.2907	499.1290	526.5995
27	551.9727	503.7191	529.1193
28	564.1239	506.2315	538.1684
29	604.4778	535.3974	543.0310
30	609.1464	557.4810	571.2865
31	631.6199	576.3386	580.8297
32	631.8592	582.3578	591.0808
33	636.9197	600.1773	595.1907
34	656.9043	603.6586	611.8670
35	660.1113	608.3716	622.7704
36	663.3100	617.0819	634.3520
37	665.4707	625.6828	637.0107
38	671.1965	629.3604	639.1276
39	680.8799	633.1861	651.6240
40	687.9900	653.5518	654.6719
41	722.4706	656.8222	669.6018
42	739.1298	681.0434	677.0293
43	740.2498	688.1236	699.644
44	748.7611	689.0752	709.2289
45	760.1307	711.2779	722.6814
46	770.4964		723.6889

cif files

***Pbca* BN**

```
_symmetry_space_group_name_H-M       'PBCA'
_symmetry_Int_Tables_number          61
_symmetry_cell_setting               orthorhombic
loop_
_symmetry_equiv_pos_as_xyz
   x,y,z
   -x+1/2,-y,z+1/2
   -x,y+1/2,-z+1/2
   x+1/2,-y+1/2,-z
   -x,-y,-z
   x+1/2,y,-z+1/2
   x,-y+1/2,z+1/2
   -x+1/2,y+1/2,z
_cell_length_a                       5.1103
_cell_length_b                       4.4336
_cell_length_c                       4.3992
_cell_angle_alpha                    90.0000
_cell_angle_beta                     90.0000
_cell_angle_gamma                    90.0000
loop_
_atom_site_label
_atom_site_type_symbol
_atom_site_fract_x
_atom_site_fract_y
_atom_site_fract_z
_atom_site_U_iso_or_equiv
_atom_site_adp_type
_atom_site_occupancy
B1   B   0.86632   0.58728   0.17112   0.00000   Uiso   1.00
N2   N   0.63460   0.09201   0.80672   0.00000   Uiso   1.00
loop_
_geom_bond_atom_site_label_1
_geom_bond_atom_site_label_2
_geom_bond_distance
_geom_bond_site_symmetry_2
_ccdc_geom_bond_type
B1   N2   1.542   2_664 S
B1   N2   1.588   4_556 S
B1   N2   1.546   7_554 S
B1   N2   1.603   8_654 S
```

```
N2      B1      1.542   2_665 S
N2      B1      1.588   4_456 S
N2      B1      1.546   7     S
N2      B1      1.603   8_646 S
```

***Pbca* AlN**

```
_symmetry_space_group_name_H-M      'PBCA'
_symmetry_Int_Tables_number          61
_symmetry_cell_setting               orthorhombic
loop_
_symmetry_equiv_pos_as_xyz
   x,y,z
   -x+1/2,-y,z+1/2
   -x,y+1/2,-z+1/2
   x+1/2,-y+1/2,-z
   -x,-y,-z
   x+1/2,y,-z+1/2
   x,-y+1/2,z+1/2
   -x+1/2,y+1/2,z
_cell_length_a                       6.1827
_cell_length_b                       5.4277
_cell_length_c                       5.2905
_cell_angle_alpha                    90.0000
_cell_angle_beta                     90.0000
_cell_angle_gamma                    90.0000
loop_
_atom_site_label
_atom_site_type_symbol
_atom_site_fract_x
_atom_site_fract_y
_atom_site_fract_z
_atom_site_U_iso_or_equiv
_atom_site_adp_type
_atom_site_occupancy
Al1   Al   0.86642   0.58540   0.17765   0.00000   Uiso   1.00
N2    N    0.63293   0.09478   0.81215   0.00000   Uiso   1.00
loop_
_geom_bond_atom_site_label_1
_geom_bond_atom_site_label_2
_geom_bond_distance
_geom_bond_site_symmetry_2
_ccdc_geom_bond_type
```

```
Al1    N2     1.917   4_556 S
Al1    N2     1.876   2_664 S
Al1    N2     1.883   7_554 S
Al1    N2     1.934   8_654 S
N2     Al1    1.917   4_456 S
N2     Al1    1.876   2_665 S
N2     Al1    1.883   7     S
N2     Al1    1.934   8_646 S
```

***Pbca* GaN**

```
_symmetry_space_group_name_H-M     'PBCA'
_symmetry_Int_Tables_number        61
_symmetry_cell_setting             orthorhombic
loop_
_symmetry_equiv_pos_as_xyz
   x,y,z
   -x+1/2,-y,z+1/2
   -x,y+1/2,-z+1/2
   x+1/2,-y+1/2,-z
   -x,-y,-z
   x+1/2,y,-z+1/2
   x,-y+1/2,z+1/2
   -x+1/2,y+1/2,z
_cell_length_a                     6.4277
_cell_length_b                     5.6022
_cell_length_c                     5.5179
_cell_angle_alpha                  90.0000
_cell_angle_beta                   90.0000
_cell_angle_gamma                  90.0000
loop_
_atom_site_label
_atom_site_type_symbol
_atom_site_fract_x
_atom_site_fract_y
_atom_site_fract_z
_atom_site_U_iso_or_equiv
_atom_site_adp_type
_atom_site_occupancy
Ga1   Ga   0.86551   0.58864   0.17995   0.00000   Uiso   1.00
N2    N    0.63323   0.09111   0.81532   0.00000   Uiso   1.00
loop_
_geom_bond_atom_site_label_1
```

_geom_bond_atom_site_label_2
_geom_bond_distance
_geom_bond_site_symmetry_2
_ccdc_geom_bond_type

Ga1	N2	1.994	4_556	S
Ga1	N2	1.943	2_664	S
Ga1	N2	1.950	7_554	S
Ga1	N2	2.012	8_654	S
N2	Ga1	1.994	4_456	S
N2	Ga1	1.943	2_665	S
N2	Ga1	1.950	7	S
N2	Ga1	2.012	8_646	S

***Pbca* InN**

_symmetry_space_group_name_H-M 'PBCA'
_symmetry_Int_Tables_number 61
_symmetry_cell_setting orthorhombic
loop_
_symmetry_equiv_pos_as_xyz
x,y,z
-x+1/2,-y,z+1/2
-x,y+1/2,-z+1/2
x+1/2,-y+1/2,-z
-x,-y,-z
x+1/2,y,-z+1/2
x,-y+1/2,z+1/2
-x+1/2,y+1/2,z
_cell_length_a 7.1722
_cell_length_b 6.2623
_cell_length_c 6.1496
_cell_angle_alpha 90.0000
_cell_angle_beta 90.0000
_cell_angle_gamma 90.0000
loop_
_atom_site_label
_atom_site_type_symbol
_atom_site_fract_x
_atom_site_fract_y
_atom_site_fract_z
_atom_site_U_iso_or_equiv
_atom_site_adp_type
_atom_site_occupancy

In1 In 0.86550 0.59022 0.18548 0.00000 Uiso 1.00
N2 N 0.63259 0.08957 0.82147 0.00000 Uiso 1.00
loop_
_geom_bond_atom_site_label_1
_geom_bond_atom_site_label_2
_geom_bond_distance
_geom_bond_site_symmetry_2
_ccdc_geom_bond_type
In1 N2 2.222 4_556 S
In1 N2 2.173 2_664 S
In1 N2 2.181 7_554 S
In1 N2 2.239 8_654 S
N2 In1 2.222 4_456 S
N2 In1 2.173 2_665 S
N2 In1 2.181 7 S
N2 In1 2.239 8_646 S

***$P4_2/mnm$* BN**

_symmetry_space_group_name_H-M 'P42/MNM'
_symmetry_Int_Tables_number 136
_symmetry_cell_setting tetragonal
loop_
_symmetry_equiv_pos_as_xyz
x,y,z
-x,-y,z
-y+1/2,x+1/2,z+1/2
y+1/2,-x+1/2,z+1/2
-x+1/2,y+1/2,-z+1/2
x+1/2,-y+1/2,-z+1/2
y,x,-z
-y,-x,-z
-x,-y,-z
x,y,-z
y+1/2,-x+1/2,-z+1/2
-y+1/2,x+1/2,-z+1/2
x+1/2,-y+1/2,z+1/2
-x+1/2,y+1/2,z+1/2
-y,-x,z
y,x,z
_cell_length_a 4.4214
_cell_length_b 4.4214
_cell_length_c 2.5483

```
_cell_angle_alpha                 90.0000
_cell_angle_beta                  90.0000
_cell_angle_gamma                 90.0000
loop_
_atom_site_label
_atom_site_type_symbol
_atom_site_fract_x
_atom_site_fract_y
_atom_site_fract_z
_atom_site_U_iso_or_equiv
_atom_site_adp_type
_atom_site_occupancy
B     B    0.32541   0.32541   0.00000   0.00000  Uiso   1.00
N2    N    0.31301  -0.31301   0.00000   0.00000  Uiso   1.00
loop_
_geom_bond_atom_site_label_1
_geom_bond_atom_site_label_2
_geom_bond_distance
_geom_bond_site_symmetry_2
_ccdc_geom_bond_type
B     N2     1.603   2_655 S
B     N2     1.603   1_565 S
B     N2     1.542   4_554 S
B     N2     1.542   4     S
N2    B      1.603   2_655 S
N2    B      1.603   1_545 S
N2    B      1.542   3_545 S
N2    B      1.542   3_544 S
```

***P4$_2$/mnm* AlN**

```
_symmetry_space_group_name_H-M     'P42/MNM'
_symmetry_Int_Tables_number        136
_symmetry_cell_setting             tetragonal
loop_
_symmetry_equiv_pos_as_xyz
  x,y,z
  -x,-y,z
  -y+1/2,x+1/2,z+1/2
  y+1/2,-x+1/2,z+1/2
  -x+1/2,y+1/2,-z+1/2
  x+1/2,-y+1/2,-z+1/2
  y,x,-z
```

-y,-x,-z
-x,-y,-z
x,y,-z
y+1/2,-x+1/2,-z+1/2
-y+1/2,x+1/2,-z+1/2
x+1/2,-y+1/2,z+1/2
-x+1/2,y+1/2,z+1/2
-y,-x,z
y,x,z
_cell_length_a 5.3203
_cell_length_b 5.3203
_cell_length_c 3.1164
_cell_angle_alpha 90.0000
_cell_angle_beta 90.0000
_cell_angle_gamma 90.0000
loop_
_atom_site_label
_atom_site_type_symbol
_atom_site_fract_x
_atom_site_fract_y
_atom_site_fract_z
_atom_site_U_iso_or_equiv
_atom_site_adp_type
_atom_site_occupancy
B Al 0.32308 0.32308 0.00000 0.00000 Uiso 1.00
N2 N 0.31540 -0.31540 0.00000 0.00000 Uiso 1.00
loop_
_geom_bond_atom_site_label_1
_geom_bond_atom_site_label_2
_geom_bond_distance
_geom_bond_site_symmetry_2
_ccdc_geom_bond_type
B N2 1.928 2_655 S
B N2 1.928 1_565 S
B N2 1.876 4_554 S
B N2 1.876 4 S
N2 B 1.928 2_655 S
N2 B 1.928 1_545 S
N2 B 1.876 3_545 S
N2 B 1.876 3_544 S

P*4$_2$/*mnm **GaN**

```
_symmetry_space_group_name_H-M       'P42/MNM'
_symmetry_Int_Tables_number          136
_symmetry_cell_setting               tetragonal
loop_
_symmetry_equiv_pos_as_xyz
   x,y,z
   -x,-y,z
   -y+1/2,x+1/2,z+1/2
   y+1/2,-x+1/2,z+1/2
   -x+1/2,y+1/2,-z+1/2
   x+1/2,-y+1/2,-z+1/2
   y,x,-z
   -y,-x,-z
   -x,-y,-z
   x,y,-z
   y+1/2,-x+1/2,-z+1/2
   -y+1/2,x+1/2,-z+1/2
   x+1/2,-y+1/2,z+1/2
   -x+1/2,y+1/2,z+1/2
   -y,-x,z
   y,x,z
_cell_length_a                       5.5473
_cell_length_b                       5.5473
_cell_length_c                       3.2214
_cell_angle_alpha                    90.0000
_cell_angle_beta                     90.0000
_cell_angle_gamma                    90.0000
loop_
_atom_site_label
_atom_site_type_symbol
_atom_site_fract_x
_atom_site_fract_y
_atom_site_fract_z
_atom_site_U_iso_or_equiv
_atom_site_adp_type
_atom_site_occupancy
B     Ga    0.32101    0.32101    0.00000    0.00000   Uiso    1.00
N2    N     0.31867   -0.31867    0.00000    0.00000   Uiso    1.00
loop_
_geom_bond_atom_site_label_1
_geom_bond_atom_site_label_2
_geom_bond_distance
```

_geom_bond_site_symmetry_2
_ccdc_geom_bond_type

B	N2	2.010	2_655 S
B	N2	2.010	1_565 S
B	N2	1.949	4_554 S
B	N2	1.949	4 S
N2	B	2.010	2_655 S
N2	B	2.010	1_545 S
N2	B	1.949	3_545 S
N2	B	1.949	3_544 S

***P*4₂*/mnm* InN**

_symmetry_space_group_name_H-M 'P42/MNM'
_symmetry_Int_Tables_number 136
_symmetry_cell_setting tetragonal
loop_
_symmetry_equiv_pos_as_xyz
x,y,z
-x,-y,z
-y+1/2,x+1/2,z+1/2
y+1/2,-x+1/2,z+1/2
-x+1/2,y+1/2,-z+1/2
x+1/2,-y+1/2,-z+1/2
y,x,-z
-y,-x,-z
-x,-y,-z
x,y,-z
y+1/2,-x+1/2,-z+1/2
-y+1/2,x+1/2,-z+1/2
x+1/2,-y+1/2,z+1/2
-x+1/2,y+1/2,z+1/2
-y,-x,z
y,x,z
_cell_length_a 6.1841
_cell_length_b 6.1841
_cell_length_c 3.6084
_cell_angle_alpha 90.0000
_cell_angle_beta 90.0000
_cell_angle_gamma 90.0000
loop_

_atom_site_label
_atom_site_type_symbol
_atom_site_fract_x
_atom_site_fract_y
_atom_site_fract_z
_atom_site_U_iso_or_equiv
_atom_site_adp_type
_atom_site_occupancy
B In 0.31801 0.31801 0.00000 0.00000 Uiso 1.00
N2 N 0.32095 -0.32095 0.00000 0.00000 Uiso 1.00
loop_
_geom_bond_atom_site_label_1
_geom_bond_atom_site_label_2
_geom_bond_distance
_geom_bond_site_symmetry_2
_ccdc_geom_bond_type
B N2 2.242 2_655 S
B N2 2.242 1_565 S
B N2 2.181 4_554 S
B N2 2.181 4 S
N2 B 2.242 2_655 S
N2 B 2.242 1_545 S
N2 B 2.181 3_545 S
N2 B 2.181 3_544 S

© 2020 by the authors. Submitted for possible open access publication under the terms and conditions of the Creative Commons Attribution (CC BY) license (http://creativecommons.org/licenses/by/4.0/).
